# Maternal cardiovascular and endothelial function from first trimester to postpartum

**DOI:** 10.1371/journal.pone.0197748

**Published:** 2018-05-21

**Authors:** Vassiliki Kolovetsiou-Kreiner, Manfred Georg Moertl, Ilona Papousek, Karin Schmid-Zalaudek, Uwe Lang, Dietmar Schlembach, Mila Cervar-Zivkovic, Helmut Karl Lackner

**Affiliations:** 1 Department of Obstetrics and Gynecology, Medical University of Graz, Graz, Austria; 2 Department of Obstetrics and Gynecology, Clinical Center, Klagenfurt, Austria; 3 Department of Psychology, Biological Psychology Unit, University of Graz, Graz, Austria; 4 Physiology, Otto Loewi Research Center, Medical University of Graz, Graz, Austria; 5 Vivantes Network of Health, Clinicum Neukoelln, Clinic of Obstetric Medicine, Berlin, Germany; 6 Department of Medical Engineering, Graz University of Technology, Graz, Austria; University of Adelaide, AUSTRALIA

## Abstract

**Objective:**

To explore noninvasively the complex interactions of the maternal hemodynamic system throughout pregnancy and the resulting after-effect six weeks postpartum.

**Methods:**

Eighteen women were tested beginning at the 12^th^ week of gestation at six time-points throughout pregnancy and six weeks postpartum. Heart rate, heart rate variability, blood pressure, pulse transit time (PTT), respiration, and baroreceptor sensitivity were analyzed in resting conditions. Additionally, hemoglobin, asymmetric and symmetric dimethylarginine and Endothelin (ET-1) were obtained.

**Results:**

Heart rate and sympathovagal balance favoring sympathetic drive increased, the vagal tone and the baroreflex sensitivity decreased during pregnancy. Relative sympathetic drive (sympathovagal balance) reached a maximum at 6 weeks postpartum whereas the other variables did not differ compared to first trimester levels. Postpartum diastolic blood pressure was higher compared to first and second trimester. Pulse transit time and endothelial markers showed no difference throughout gestation. However, opposing variables PTT and asymmetric dimethylarginine (ADMA) were both higher six weeks postpartum.

**Conclusions:**

The sympathetic up regulation throughout pregnancy goes hand in hand with a decreased baroreflex sensitivity. In the postpartum period, the autonomic nervous system, biochemical endothelial reactions and PTT show significant and opposing changes compared to pregnancy findings, indicating the complex aftermath of the increase of blood volume, the changes in perfusion strategies and blood pressure regulation that occur in pregnancy.

## Introduction

The course of a normal pregnancy is characterized by several profound changes of the maternal cardiovascular system in order to adapt blood pressure to the needs of pregnancy and prepare for delivery. These changes include increases in maternal cardiac output, heart rate, and intravascular volume, and are accompanied by a decreased vascular resistance, mainly due to a reduction of the total peripheral resistance and an increase of the vascular compliance [1–4]. Most of these changes resolve by 6 weeks to 3 months after delivery [5]. The autonomic nervous system, which leads to a shift towards a lower sympathetic and a higher vagal modulation during the 1^st^ trimester and a continuing shift towards a higher sympathetic and lower vagal modulation at late pregnancy collaborates with biochemical factors, such as the Asymmetric Dymethylarginine-Nitric Oxide- Pathway (ADMA-NO-Pathway) or the NO-antagonist endothelin-1 (ET-1) secreted by the endothelium to regulate these changes [6–11].

The mechanisms of biochemical and physiological interactions during normal pregnancy are however still not fully understood and are topic of ongoing debates.

Development of non-invasive methods which allow longitudinal analysis of cardiovascular changes throughout and in the time period after pregnancy and parallelization of these findings with endothelial alterations is of great value in understanding the physiology and further on pathophysiology (e.g. complications like preeclampsia, IUGR, etc.) of cardiovascular adaptations in pregnancy.

This prospective study aimed to assess cardiovascular changes in women with uncomplicated pregnancies. We evaluated the influence of the autonomic nervous system and the ADMA-NO-Endothelial-System by analyzing heart rate (HR), the ratio between low and high frequency components (LF/HF ratio) of HRV spectra, systolic and diastolic blood pressure (SBP, DBP), pulse transit time (PTT), thoracic impedance (Z_0_) and baroreflex sensitivity (BRS) and parallelizing these findings with serum levels of endothelin (ET-1), asymmetric dimethylarginine (ADMA), symmetric dimethylarginine (SDMA) and hemoglobin (Hb). Postpartum application of these tests (6 weeks p.p.) was performed in order to describe conversion of these changes to a non-pregnant state.

## Material and methods

### Ethics statement

The study was performed in accordance with the 1964 Declaration of Helsinki and was approved by the Ethics Committee of the Medical University of Graz. Written informed consent was obtained from all participants.

### Participants

Pregnant women who underwent first trimester screening were asked to participate in the study concerning cardiovascular alterations throughout pregnancy. All women gave written informed consent to participate in the study performed at the Department of Obstetrics and Gynecology, Medical University of Graz. Women with pre-existing diseases such as insulin-dependent diabetes or cardiovascular or renal diseases, and/or pregnancy related complications and disorders such as preeclampsia were excluded from the study. For this study, we analyzed the subgroup of 18 women with uneventful pregnancies, deliveries and healthy newborns. All 18 women attended all seven scheduled visits throughout pregnancy and the postpartum visit, which occurred 6 weeks after delivery. They had singleton pregnancies, did not receive any medication throughout pregnancy except of Vitamin-Supplements and all women were non-smokers.

### Experimental procedure

After participants were familiarized with the test protocol, equipment and personnel, electrodes were attached and patients were positioned in the 15° left lateral position, ensuring a continuous venous blood flow to the heart. During the whole procedure the participants were asked not to talk or make abrupt movements. The study protocol consisted of a 20 min adaptation period followed by 10 min recording at rest. For analysis a five minute epoch commencing at 4 min of rest was used.

Measurements were performed at seven visits longitudinally during pregnancy at time intervals of 4–5 weeks and one last visit six weeks postpartum (*visit 1*: range 11^+3^–13^+1^, *visit 2*: 15^+0^–18^+4^, *visit 3*: 19^+3^–23^+4^, *visit 4*: 23^+6^–26^+3^, *visit 5*: 29^+1^–32^+1^, *visit 6*: 33^+4^–36^+4^, *visit 7*: 37^+4^–41^+2^, and *postpartum*: 4^+6^–9^+3^).

### Data acquisition and preprocessing

Continuous monitoring of blood pressure (BP,sampling rate, sr = 100Hz, BP_range_ = 50–250 mmHg, ±5 mmHg), R-R-Interval (RRI, 3-lead electrocardiography, sr = 1 kHz, f_cut-off_ = 0.08–150 Hz) and thoracic impedance were carried out with the Task Force^®^ Monitor (TFM^®^; CNSystems, Medizintechnik AG, Graz, Austria) [12]. Continuous BP was derived from the finger using a refined version of the vascular unloading technique and corrected to absolute values with oscillometric BP measurement by the TFM^®^ [12]. Electrodes were placed at the neck and thoracic regions, the latter specifically at the midclavicular line at the xiphoid process level.

To obtain RRI time series with equidistant time steps, the beat-to-beat values were resampled at 4 Hz, using piecewise cubic spline interpolation after artifact correction. Single artifacts were replaced by interpolation and its appearance recorded [13].

Furthermore, the respiratory signal was derived from the thoracic impedance. All five minute epochs fulfilled the criteria of at least 95% valid R-R intervals (RRI).

Time domain indexes of heart rate variability (HRV) were computed as the standard deviation of normal-to-normal beat (SDNN) and root mean squared successive differences (RMSSD) of R-R intervals. SDNN reflects sympathetic and to some extent vagal tone whereas RMSSD reflects the vagal tone only.

For frequency domain indexes of RRI we used Fast Fourier Transform with a Hanning window for spectral analysis of cardiovascular signals on the blocks of 5 min epochs, after resampling and removing the trend of 2^nd^ order. Low frequency (LF) was defined as 0.04–0.15 Hz, high frequency (HF) was defined as 0.15–0.40 Hz, according to published recommendations [14]. Because of skewed distributions of frequency domain indexes, a natural logarithmic transformation was applied (ln(LF), ln(HF) and the LF/HF ratio ln(LF/HF)). The variable ln(LF) includes mainly sympathetic, ln(HF) vagal influences, and ln(LF/HF) is considered to mirror the sympathovagal balance, that is the autonomic state.

The pulse transit time (PTT) was calculated by the time difference between the r-peak of QRS-complex of the ECG signal (Einthoven lead II) and the point with the maximal slope of the pulse wave detected at the fingertip during the systolic rise.

The sequence technique was used for the assessment of baroreceptor reflex sensitivity (BRS) [15]. Usually, sequences of three to six consecutive cardiac beats are sought in which an increase in SBP is accompanied by an increase in RRI, or in which a decrease in SBP is accompanied by a decrease in RRI. The regression line between the SBP and the RRI values produces an estimate of BRS. An equivalent change in RRI and SBP for at least three consecutive cardiac cycles was defined as a regulatory event if the following criteria were fulfilled: (1) RRI variations > 4 ms; (2) SBP changes > 1 mmHg.

ADMA and SDMA and Endothelin-1 were determined by ELISA Assay Kits. Antigen is bound to the solid phase of the microtiter plate. Antigen in the samples is acylated and competes with solid phase bound antigen for a fixed number of antiserum binding sites. When the system is in equilibrium, free antigen and free antigen-antiserum complexes are removed by washing. The antigens bound to the solid phase are detected by antibodies. The amount of antibody bound to the solid phase antigen is inversely proportional to the antigen concentration of the sample.

Data are presented as mean ± standard deviation. To evaluate the effects of pregnancy repeated measures analyses of variance (ANOVAs) were conducted with *“visit”* (seven time points; within-subjects factor) as independent variable. If necessary, Greenhouse-Geisser corrections were used to adjust for non-sphericity of the variance–covariance matrices.

To evaluate the difference between course of pregnancy and postpartum values Bonferroni corrected planned multiple-comparisons (*t*-tests) were conducted (*visit 1*, *visit 4*, *visit 6*, and *visit 7* representing trimester 1, trimester 2, trimester 3, and immediately before term).

## Results

Data presented here are from 18 pregnant women of age 32 ± 4 years (mean ± SD; range: 25–38 years), height 166 ± 5 cm (158–177 cm), weight 61 ± 8 kg (47–77 kg before pregnancy), and body mass index (BMI) 22.3 ± 2.3 kg/m^2^ (18.8–26.4 kg/m^2^). Body weight increased to 75 ± 8 kg (62–88 kg) and BMI to 27.4 ± 2.3 kg/m^2^ (23.5–32.0 kg/m^2^) at term, full term was at 279 ± 6 days (266–291 days). 10 women had no history of previous gestations, 3 women were coursing the second gestation, and 5 women had history of more than two gestations. 12 out of the 18 healthy newborns were female, the birth weight was on average 3291 ± 328 g (mean ± SD; range: 2660 – 3800g), the birth height was 51 ± 2 cm (48–54 cm).

### Cardiovascular and hemodynamic variables

#### Heart rate and heart rate variability variables

The ANOVAs yielded a significant main effect of *visit* for heart rate, RMSSD, ln(HF) and ln(LF/HF) but did not reach significance level for SDNN and ln(LF).

Heart rate and ln(LF/HF) increased throughout gestation, indicated by a linear trend (*F*(1,17) = 8.5, *p* < .01, *η*_p_^2^ = 0.33, *F*(1,17) = 16.9, *p* = .001, *η*_p_^2^ = .50, respectively) as well as and quadratic trend (*F*(1,17) = 24.8, *p* < .001, *η*_p_^2^ = 0.59; *F*(1,17) = 9.0, *p* < .01, *η*_p_^2^ = 0.35). RMSSD (linear trend: *F*(1,17) = 6.8, *p* < .05, *η*_p_^2^ = .29, quadratic trend: *F*(1,17) = 21.0, *p* < .001, *η*_p_^2^ = .55) and ln(HF) (linear trend: *F*(1,17) = 13.0, *p* = .002, *η*_p_^2^ = .43, quadratic trend: *F*(1,17) = 18.5, *p* < .001, *η*_p_^2^ = .52) decreased with advancing gestational age (see S1 Fig).

No difference between *visit 1* and *postpartum* were observed for heart rate, SDNN, RMSSD, and ln(HF), whereas ln(LF/HF) was significantly higher *postpartum* (*t*_17_ = 5.4, *p* < .001). Furthermore, ln(LF) at *postpartum* was higher compared to *visit 1*, *visit 4*, *visit 6*, and *visit 7 (t*_17_ = 3.3, *p* < .01, *t*_17_ = 5.2, *p* < .001, *t*_17_ = 5.7, *p* < .001, and *t*_17_ = 4.7, *p* < .001). Means ± SD as well as the *F*-statistics and the subsequently performed *t*-tests are reported in Table 1.

**Table 1:** Heart rate and heart rate variability variables (mean ± SD) of participants.

	*week 11–13*	*week 15–18*	*week 19–23*	*week 24–27*	*week 29–32*	*week 33–36*	*week 37–41*	*postpartum*	*F-Statistics* (throughout gestation)
***HR (bpm)***									
	74.5 ± 9.1	78.9 ± 9.8	81.1 ± 8.1	82.4 ± 9.4	84.6 ± 9.5	84.4 ± 10.8	78.2 ± 7.4	72.2 ± 11.5^b^^,^^c^	*F*_(6,102)_ = 8.3, ε = 0.69, p < .001, η_p_^2^ = .33
***SDNN (ms)***									
	50.4 ± 20.9	41.0 ± 22.3	41.2 ± 15.2	36.1 ± 12.6	37.8 ± 15.0	39.8 ± 15.6	45.7 ± 18.9	60.1 ± 28.6^b^	*F*_(6,102)_ = 2.4, ε = 0.49, p < .08, η_p_^2^ = .12
***RMSSD (ms)***									
	42.4 ± 29.8	29.7 ± 23.7	25.9 ± 18.0	21.3 ± 11.9	21.9 ± 18.8	20.0 ± 10. 7	27.9 ± 12.2	42.3 ± 27.9^b^^,^^c^	*F*_(6,102)_ = 7.5, ε = 0.41, p = .001, η_p_^2^ = .31
***ln(LF) (ms***^***2***^***)***									
	6.19 ± 0.69	5.77 ± 1.00	5.82 ± 0.76	5.71 ± 0.73	5.72 ± 0.76	5.81 ± 0.80	6.06 ± 0.74	6.94 ± 0.98^a^^,^^b^^,^^c^^,^^d^	*F*_(6,102)_ = 2.1, ε = 0.76, p = .08, η_p_^2^ = .11
***ln(HF) (ms***^***2***^***)***									
	5.97 ± 1.29	5.24 ± 1.32	5.14 ± 1.26	4.86 ± 0.98	4.78 ± 1.19	4.81 ± 1.09	5.30 ± 0.97	5.81 ± 1.37^b^^,^^c^	*F*_(6,102)_ = 7.3, ε = 0.75, p < .001, η_p_^2^ = .30
***ln(LF/HF) (-)***									
	0.21 ± 0.83	0.53 ± 0.85	0.69 ± 0.91	0.84 ± 0.69	0.94 ± 0.81	1.00 ± 0.67	0.75 ± 0.78	1.12 ± 0.67^a^	*F*_(6,102)_ = 5.0, ε = 0.75, p = .002, η_p_^2^ = .23

HR = heart rate; SDNN = standard deviation of normal-to-normal beat; RMSSD = root mean squared successive differences of R-R intervals; LF = low frequency (0.04–0.15Hz); HF = low frequency (0.15–0.40Hz); ln = natural logarithmic transformation.

Superscript letters denote significant differences between *postpartum* and

^a^week 11–13

^b^week 24–27

^c^week 33–36, and

^d^week 37–41, respectively.

week 11–13 refers to *visit 1* (range: 11^+3^–13^+1^), week 15–18: *visit 2* (15^+0^–18^+4^), week 19–23: *visit 3* (19^+3^–23^+4^), week 24–27: *visit 4 (*23^+6^–26^+3^), week 29–32: *visit 5* (29^+1^–32^+1^), week 33–36: *visit 6*: (33^+4^–36^+4^), week 37–41: *visit 7* (37^+4^–41^+2^), postpartum: six weeks postpartum (range: 4^+6^–9^+3^).

The LF/HF ratio as a measure of characterizing the autonomic state resulting from sympathetic and parasympathetic influences increases with advancing gestational age, whereby six weeks postpartum it did not return to the level of the end of the first trimester.

#### Blood pressure variables

Systolic and diastolic blood pressure were significantly influenced by gestation and increased during gestation (SBP, linear trend: *F*(1,17) = 16.3, *p* = .001, *η*_p_^2^ = 0.49, quadratic trend: *F*(1,17) = 9.1, *p* < .01, *η*_p_^2^ = 0.35; DBP, linear trend: *F*(1,17) = 7.5, *p* < .05, *η*_p_^2^ = 0.31). No difference throughout gestation was seen for pulse transit time. However, pulse transit time at *postpartum* was higher compared to *visit 1*, *visit 4*, *visit 6*, and *visit 7 (t*_17_ = 3.3, *p* < .01, *t*_17_ = 5.2, *p* < .001, *t*_17_ = 5.7, *p* < .001, and *t*_17_ = 4.7, *p* < .001; see S1 Fig). Means ± SD as well as the *F*-statistics and the subsequently performed *t*-tests are reported in Table 2.

**Table 2:** Blood pressure and pulse transit time variables (mean ± SD) of participants.

	*week 11–13*	*week 15–18*	*week 19–23*	*week 24–27*	*week 29–32*	*week 33–36*	*week 37–41*	*postpartum*	*F-Statistics* (throughout gestation)
***SBP (mmHg)***									
	103.8 ± 10.5	101.9 ± 13.8	104.2 ± 10.0	101.4 ± 13.7	106.7 ± 15.6	108.6 ± 13.5	111.2 ± 13.6	109.8 ± 13.5^b^	*F*_(6,102)_ = 3.1, p < .05, η_p_^2^ = .15
***DBP (mmHg)***									
	62.5 ± 9.7	61.4 ± 11.6	64.0 ± 9.7	61.8 ± 11.1	66.5 ± 13.0	66.8 ± 11.3	68.8 ± 13.9	71.2 ± 11.8^a^^,^^b^	*F*_(6,102)_ = 2.7, p < .05, η_p_^2^ = .14
***PTT (ms)***									
	249 ± 10	250 ± 11	250 ± 12	251 ± 11	253 ± 13	250 ± 12	252 ± 10	269 ± 16^a^^,^^b^^,^^c^^,^^d^	*F*_(6,102)_ = 0.8, ns.

SBP = systolic blood pressure; DBP = diastolic blood pressure; PTT = pulse transit time.

Superscript letters denote significant differences between *postpartum* and

^a^week 11–13

^b^week 24–27

^c^week 33–36, and

^d^week 37–41, respectively.

week 11–13 refers to *visit 1* (range: 11^+3^–13^+1^), week 15–18: *visit 2* (15^+0^–18^+4^), week 19–23: *visit 3* (19^+3^–23^+4^), week 24–27: *visit 4 (*23^+6^–26^+3^), week 29–32: *visit 5* (29^+1^–32^+1^), week 33–36: *visit 6*: (33^+4^–36^+4^), week 37–41: *visit 7* (37^+4^–41^+2^), postpartum: six weeks postpartum (range: 4^+6^–9^+3^).

#### Thoracic impedance, respiration and BRS

No difference of thoracic impedance and respiratory frequency were observed during the course of pregnancy whereas ΔZ_0,Resp_ , the respiratory related change of the thoracic impedance indicating the tidal volume, was significantly influenced by gestation. ΔZ_0,Resp_ increased during the course of pregnancy denoted by a linear trend (*F*(1,17) = 24.4, *p* < .001, *η*_p_^2^ = .59). The subsequently performed *t*-tests showed no difference between *visit 1* and *postpartum* (*t*_17_ = -1.0, *ns*.). The BRS decreased during the course of pregnancy indicated by a linear (*F*(1,17) = 12.4, *p* < .01, *η*_p_^2^ = .42) and quadratic trend (*F*(1,17) = 14.2, *p* < .01, *η*_p_^2^ = .45). A significant difference for BRS could be shown between advancing gestational age and *postpartum* could be observed for *visit 3* only (*t*_17_ = 3.5, *p* < .01). Means ± SD as well as the *F*-statistics and the *t*-tests are reported in Table 3.

**Table 3:** Thoracic impedance variables and baroreflex sensitivity (mean ± SD) of participants.

	*week 11–13*	*week 15–18*	*week 19–23*	*week 24–27*	*week 29–32*	*week 33–36*	*week 37–41*	*postpartum*	*F-Statistics* (throughout gestation)
***Z***_***0***_ ***(Ohm)***									
	34.7 ± 3.8	34.8 ± 4.7	35.5 ± 3.5	36.5 ± 4.6	36.4 ± 3.2	34.6 ± 4.0	33.8 ± 4.6	35.6 ± 3.4	*F*_(6,102)_ = 2.1, *ns*.
**Δ*Z***_***0*,*Resp***_ ***(Ohm)***									
	0.40 ± 0.11	0.45 ± 0.19	0.47 ± 0.14	0.50 ± 0.08	0.59 ± 0.22	0.63 ± .019	0.61 ± 0.25	0.37 ± 0.09^b^^,^^c^^,^^d^	*F*_(6,102)_ = 10.1, ε = 0.54, p < .001, η_p_^2^ = .37
***RF (min***^***-1***^***)***									
	19.4 ± 3.3	18.8 ± 3.4	19.3 ± 3.2	18.8 ± 2.5	18.1 ± 2.8	18.1 ± 2.7	18.1 ± 2.7	17.3 ± 2.5	*F*_(6,102)_ = 2.0, *ns*.
***BRS (ms/mmHg)***									
	19.8 ± 11.5	15.4 ± 11.9	13.8 ± 7.8	12.0 ± 5.6	11.0 ± 9.6	10.2 ± 5.2	12.3 ± 5.3	17.8 ± 11.5^c^	*F*_(6,102)_ = 7.5, ε = 0.46, p < .001, η_p_^2^ = .31

ΔZ_0,RESP_ = change of thoracic impedance (Z_0_) driven by respiration; RF = respiratory frequency; BRS = baroreflex sensitivity.

Superscript letters denote significant differences between *postpartum* and

^a^week 11–13

^b^week 24–27

^c^week 33–36, and

^d^week 37–41, respectively.

week 11–13 refers to *visit 1* (range: 11^+3^–13^+1^), week 15–18: *visit 2* (15^+0^–18^+4^), week 19–23: *visit 3* (19^+3^–23^+4^), week 24–27: *visit 4 (*23^+6^–26^+3^), week 29–32: *visit 5* (29^+1^–32^+1^), week 33–36: *visit 6*: (33^+4^–36^+4^), week 37–41: *visit 7* (37^+4^–41^+2^), postpartum: six weeks postpartum (range: 4^+6^–9^+3^).

### Laboratory findings

Hemoglobin showed significant changes and decreased, indicated by a linear and quadratic trend (*F*(1,17) = 4.6, *p* < .05, *η*_p_^2^ = 0.21; *F*(1,17) = 24.9, *p* < .001, *η*_p_^2^ = 0.59), during gestation. No difference throughout gestation were seen for ET-1, ADMA and SDMA.

Six weeks postpartum a significant higher ADMA value were observed compared to ADMA throughout gestation (*postpartum* compared to *visit 1*: *t*_17_ = 3.4, *p* < .01; to *visit 4*: *t*_17_ = 6.1, *p* < .001, to *visit 6*: *t*_17_ = 4.4, *p* < .001, and to *visit 7*: *t*_17_ = 5.0, *p* < .001; see S1 Fig). No differences were observed for hemoglobin, endothelin and SDMA. Means ± SD as well as the *F*-statistics and the subsequently performed *t*-tests are reported in Table 4.

**Table 4:** Laboratory values (mean ± SD) of participants.

	*week 11–13*	*week 15–18*	*week 19–23*	*week 24–27*	*week 29–32*	*week 33–36*	*week 37–41*	*postpartum*	*F-Statistics* (throughout gestation)
***Hb (g/dl)***									
	13.3 ± 1.2	13.0 ± 1.0	12.7 ± 0.9	12.6 ± 0.9	12.4 ± 0.8	12.5 ± 0.8	13.0 ± 0.8	13.4 ± 1.2	*F*_(6,102)_ = 5.4, p = .002, η_p_^2^ = .24
**ET-1 *(fmol/ml)***									
	0.65 ± 0.72	0.60 ± 0.62	0.65 ± 0.61	0.59 ± 0.67	0.53 ± 0.51	0.51 ± 0.39	0.62 ± 0.57	0.90 ± 0.96	*F*_(6,102)_ = 0.8, ns.
***ADMA (*μ*mol/l)***									
	0.41 ± 0.09	0.39 ± 0.08	0.36 ± 0.09	0.37 ± 0.11	0.40 ± 0.07	0.42 ± 0.07	0.41 ± 0.08	0.56 ± 0.12^a^^,^^b^^,^^c^^,^^d^	*F*_(6,102)_ = 1.5, ns.
***SDMA (*μmol*/l)***									
	0.43 ± 0.09	0.41 ± 0.10	0.39 ± 0.11	0.41 ± 0.12	0.39 ± 0.10	0.39 ± 0.08	0.38 ± 0.11	0.39 ± 0.06	*F*_(6,102)_ = 0.8, ns.

Hb = hemoglobin, Endo = endothelin, ADMA = asymmetric dimethylarginine, SDMA = symmetric dimethylarginine.

Superscript letters denote significant differences between *postpartum* and

^a^week 11–13

^b^week 24–27

^c^week 33–36, and

^d^week 37–41, respectively.

week 11–13 refers to *visit 1* (range: 11^+3^–13^+1^), week 15–18: *visit 2* (15^+0^–18^+4^), week 19–23: *visit 3* (19^+3^–23^+4^), week 24–27: *visit 4 (*23^+6^–26^+3^), week 29–32: *visit 5* (29^+1^–32^+1^), week 33–36: *visit 6*: (33^+4^–36^+4^), week 37–41: *visit 7* (37^+4^–41^+2^), postpartum: six weeks postpartum (range: 4^+6^–9^+3^).

## Discussion

This study reports about comprehensive changes in maternal cardiovascular hemodynamics during pregnancy and the effects of these changes, respectively their remodeling to a normal, non-pregnant status six weeks postpartum. Our study showed that a sympathetic up regulation throughout pregnancy and in puerperium goes hand in hand with decreased baroreflex sensitivity. Pulse transit time and biochemical markers (ADMA, SDMA and Endothelin-1), which represent the endothelial function, did not differ during normal pregnancies in our collective. However, in postpartum period, the autonomic nervous system, biochemical endothelial reactions and pulse transit time show significant changes compared to pregnancy findings.

### Heart rate and heart rate variability variables

Heart rate (HR) linearly increases throughout pregnancy and returns to levels of early gestation at 6 weeks postpartum, in line with previous studies [4,8,16,17].

Heart rate variability was described in our study by its time domain indexes SDNN and RMSSD. Both of them showed similar levels at the beginning of pregnancy and 6 weeks postpartum, whereas RMSSD which describes the beat-to-beat changes of the heart rate decreased with advancing gestation. This is explained by the shortened R-R intervals due to the increasing frequency of the maternal heart rate [18–20]. Ln(HF) decreases with advancing gestational age and ln(LF/HF), which characterizes the autonomic state of the maternal system, increases during the course of pregnancy reflecting the up-regulation of the cardiac output. These findings are in agreement with research using sophisticated nonlinear analyzing approaches in addition to conventional linear methods of HRV [21, 22]. Walther et al. [23] found significantly restricted HRV in pregnancy with a clear trend of reduced HRV even four days after delivery and concluded that the maternal cardiovascular system was still strongly affected by pregnancy at that time. In our study, the observation interval after delivery was much more extended. ln(LF/HF) reached significantly higher levels 6 weeks postpartum compared to during pregnancy, and remained higher compared to the end of the first trimester (Visit1), mainly due to higher levels of ln(LF) postpartum compared to pregnancy levels. Hence, our findings add to the literature using longitudinal study designs to analyze cardiovascular variability postpartum [24, 25].

### Pulse transit time

Pulse transit time (PTT) is the time (in ms) needed for a cardiac signal to travel through the vascular tree to distant locations [26]. Conditions that influence PTT are heart rate, blood pressure and vascular tone. Considering that the augmentation index (AIx), as a marker of arterial stiffness decreased from the first to the second trimester reaching a nadir in mid-pregnancy and increased again at the third trimester, an increase in PTT would be expected, though AIx and PWV cannot be used as direct indexes of arterial stiffness because of the different information they contain [27]. PTT-values during pregnancy have been topic of investigation in recent years, with quite contradictory results. Increased, decreased and almost constant values have been reported [28–30]. However, recent research showed that PTT has the potential to be used as reference for respiratory-related variations of heart rate [31]. In our study the respiratory frequency remained almost constant, and PTT showed no alterations throughout pregnancy. A potential limitation is that we measured PTT at the upper extremity, which is actually not compatible with the general vascular stiffness. However, PTT is prolonged 6 weeks p.p., reaching levels higher than at the beginning and end of pregnancy (visits 1,4,6 and 7). This might occur due to the complex aftermath of changes in blood volume and blood pressure during pregnancy.

### Respiration and baroreflex sensitivity

The primary role of the arterial baroreflex is the immediate and short-term adjustment of BP following perturbations around an existing mean pressure [32]. Baroreflex sensitivity (BRS) was reported to decrease from term to the postpartum period in normotensive pregnancy [32–35]. Valdes et al. [36] demonstrated a progressive blunting of the heart rate response to tilt as pregnancy progressed, suggesting a decrease in BRS in late pregnancy. In our population BRS decreased throughout pregnancy, which might be related to a reduction of vagal tone, rather than an increase in sympathetic activity. However, thoracic impedance and respiratory frequency remained almost constant throughout pregnancy in our population of healthy women.

### Laboratory findings

The endothelium plays a crucial role in the maintenance of vascular tone and structure [37]. In the early 1990s NOS-System was first described [38,39]. NO as well as its endogenous inhibitor ADMA became subjects of investigation mainly in order to find explanations for pregnancies complicated by preeclampsia.

ET-1 is produced by different female reproductive tissues, including uterus [40] where ET-1 regulates pleiotropic cell functions associated with physiology and pathology of reproduction. The role of the ET-1 axis in female reproductive disorders is now well established. It is known to be the most powerful vasoconstrictor [41].

In our collective ET-1 levels did not show any significant alterations throughout pregnancy and in the postpartum period. In contrast, Ajne et al. reported that plasma levels of endothelin-1 decrease in the second trimester during normal pregnancy [42]

Higher levels of ADMA in the second and third trimester of pregnancy were in patients with preeclampsia [43]. ADMA was almost constant throughout pregnancy in our data but showed a significant increase 6 weeks postpartum. Stable levels of ADMA during pregnancy were reported by others (9), while Rizos et al. [44] and Holden et al. [45] found increasing ADMA between the 1^st^ and 3^rd^ trimester.

Valtonen et al. [46] and Slaghekke et al. [47] showed a reduction of ADMA during early pregnancy compared to non-pregnant women, leading to an increase of NO and an enhanced placental perfusion. As we started collecting blood samples at the 12^th^ week of gestation, our study did not provide information on early pregnancy. We assume that the increased ADMA 6 weeks postpartum compared to pregnancy levels represents a reactive feedback of ADMA to “normal”, “non-pregnant” levels.

Symmetric dimethylarginine (SDMA), is a structural ADMA isomer, which does not interfere with NOS, but it is a competitor of L-arginine transport, and may therefore interfere with NO production and endothelial function [48]. Little is known about SDMA levels in normal pregnancies. In our collective of uncomplicated pregnancies SDMA did not show alterations. Levels where similar to gestational levels in the postpartum period.

In summary, the results of our study are consistent with and extended the findings of previous studies. The strengths and specialities of this study are the stringent inclusion and exclusion criteria, the thorough cardiovascular evaluation of the pregnant women, and the observation window of approximately 36 weeks (12^th^ week of gestation until 6 weeks postpartum).

However, the small sample size and the observational nature of the study should also be considered as an important limitation of this study. The latter make it impossible to control for many potential confounders. Some of them have, however, been analyzed and accounted for as much as possible. One further limitation concerning the measuring-technique is that pulse wave velocity over the upper extremity does not fully comply with central pulse wave velocities, nor with those at the uterine circulation.

We do, nevertheless, acknowledge that observations from this small study provide rationale for future activity in several areas of cardiovascular analyses in pregnancy. If replicated, the findings will contribute to our understanding of the causality of cardiovascular changes throughout pregnancy and the role of the endothelium in that causality.

## Conclusion

The cardiovascular changes during pregnancy follow expected patterns. The autonomic nervous system as well as biochemical reactions in the endothelium are contributing in regulating these changes.

The increase of the sympathetic regulation is followed by a decrease of baroreflex sensitivity throughout pregnancy. In puerperium the autonomic nervous system and endothelial reactions are struggling to achieve a “normal” non-pregnant state again. Nonetheless, the autonomic nervous system, biochemical endothelial reactions and PTT show significant and opposing changes compared to pregnancy findings, indicating the complex aftermath of the increase of blood volume, the changes in perfusion strategies, and blood pressure regulation that occur in pregnancy. The findings underpin the value of integrating the puerperium in the analysis of pregnancy-related cardiovascular changes.

## Supporting information

S1 Fig Longitudinal graphical representation for selected variables. Longitudinal changes of heart rate (A), low frequency of heart rate variability spectra (ln(LF); B), pulse transit time (C) and asymmetric dimethylarginine (ADMA); ^a,b,c,d^ denotes significant differences between *postpartum* and week 11–13 (^a^), week 24–27 (^b^), week 33–36 (^c^), and week 37–41 (^d^), respectively. week 11–13 refers to *visit 1* (range: 11^+3^–13^+1^), week 15–18: *visit 2* (15^+0^–18^+4^), week 19–23: *visit 3* (19^+3^–23^+4^), week 24–27: *visit 4 (*23^+6^–26^+3^), week 29–32: *visit 5* (29^+1^–32^+1^), week 33–36: *visit 6*: (33^+4^–36^+4^), week 37–41: *visit 7* (37^+4^–41^+2^), postpartum: six weeks postpartum (range: 4^+6^–9^+3^).
(TIF)

S1 File List of abbreviations. (PDF)

S1 Table Summary overview of results related to Tables 1–4. **↑**: significant increase of related variable throughout pregnancy or postpartum (planned multiple-comparisons), respectively; **↓**: significant decrease of related variable throughout pregnancy or postpartum, respectively; **↔**: no significant change of related variable throughout pregnancy or postpartum, respectively.
(PDF)

S2 Table Dataset of the reported data. (XLS)
