# Supplementary figures and images for: Maternal cardiovascular and endothelial function from first trimester to postpartum
Source: PLoS One. 2018 May 21;13(5):e0197748. doi: 10.1371/journal.pone.0197748 (PMC5962097; doi:10.1371/journal.pone.0197748)

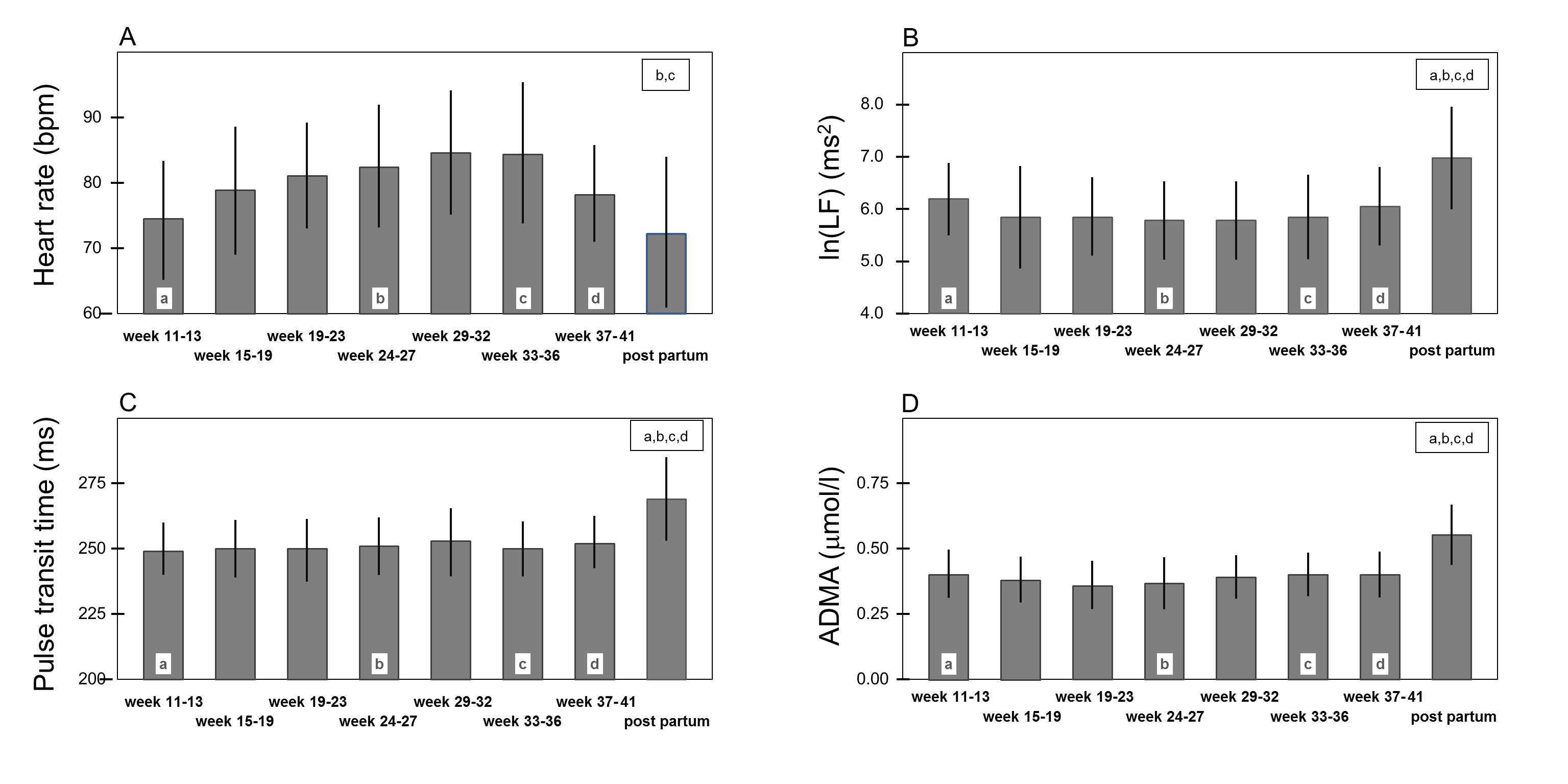

Supplement: S1 Fig — Longitudinal changes of heart rate (A), low frequency of heart rate variability spectra (ln(LF); B), pulse transit time (C) and asymmetric dimethylarginine (ADMA); a,b,c,d denotes significant differences between postpartum and week 11–13 (a), week 24–27 (b), week 33–36 (c), and week 37–41 (d), respectively. week 11–13 refers to visit 1 (range: 11+3–13+1), week 15–18: visit 2 (15+0–18+4), week 19–23: visit 3 (19+3–23+4), week 24–27: visit 4 (23+6–26+3), week 29–32: visit 5 (29+1–32+1), week 33–36: visit 6: (33+4–36+4), week 37–41: visit 7 (37+4–41+2), postpartum: six weeks postpartum (range: 4+6–9+3). (TIF) [file pone.0197748.s001.tif]
