# Supplementary material for: Maternal cardiovascular and endothelial function from first trimester to postpartum
Source: PLoS One. 2018 May 21;13(5):e0197748. doi: 10.1371/journal.pone.0197748 (PMC5962097; doi:10.1371/journal.pone.0197748)
Supplement: S1 File — (PDF) [file pone.0197748.s002.pdf]

S1 File. List of abbreviations.

sr	Sampling rate
HR	Heart Rate
RRI	R-R-Interval
HRV	Heart rate variability
SDNN	Standard deviation of normal to normal beat interval
RMSSD	Square root of the mean squared di□erences of successive normal to normal beat intervals
LF	Low frequency spectral component of HRV (0.04-0.15 Hz)
HF	High frequency spectral component of HRV (0.15-0.40 Hz)
LF/HF-ratio	Ratio between low and high frequency components (LF/HF ratio) of HRV spectra
ln	natural logarithmic transformation
BP	Blood pressure
SBP	Systolic blood pressure
DBP	Diastolic blood pressure
BRS	Baroreceptor reflex sensitivity
RF	Respiratory frequency
PTT	Pulse transit time
Z_0	Thoracic impedance
$\Delta Z_{0,Resp}$	Change of thoracic impedance driven by respiration;
Hb	Hemoglobin
ET-1	Endothelin 1
ADMA	Asymmetric dimethylarginine
SDMA	Symmetric dimethylarginine
