# Supplementary material for: Maternal cardiovascular and endothelial function from first trimester to postpartum
Source: PLoS One. 2018 May 21;13(5):e0197748. doi: 10.1371/journal.pone.0197748 (PMC5962097; doi:10.1371/journal.pone.0197748)
Supplement: S1 Table — ↑: significant increase of related variable throughout pregnancy or postpartum (planned multiple-comparisons), respectively; ↓: significant decrease of related variable throughout pregnancy or postpartum, respectively; ↔: no significant change of related variable throughout pregnancy or postpartum, respectively. (PDF) [file pone.0197748.s003.pdf]

S1 Table. Summary overview of results related to table 1- 4.

	throughout pregnancy	postpartum
**Heart rate and heart rate variabiltiy variables**		
HR	↑	↓
SDNN	↔	↑
RMSSD	↓	↑
ln(LF)	↔	↑
ln(HF)	↓	↑
ln(LF/HF)	↑	↑
**Blood pressure variables**		
SBP	↑	↑
DBP	↑	↑
PTT	↔	↑
**Thoracic impedance, respiration and BRS**		
Z_0	↔	↔
$\Delta Z_{0,Resp}$	↑	↓
RF	↔	↔
BRS	↓	↑
**Laboratory findings**		
Hb	↓	↔
ADMA	↔	↑
SDMA	↔	↔
ET-1	↔	↔
